# Agrin-Lrp4 pathway in hippocampal astrocytes restrains development of temporal lobe epilepsy through adenosine signaling

**DOI:** 10.1186/s13578-024-01241-5

**Published:** 2024-05-23

**Authors:** Zi-Yang Liu, Yuan-Quan Li, Die-Lin Wang, Ying Wang, Wan-Ting Qiu, Yu-Yang Qiu, He-Lin Zhang, Qiang-Long You, Shi-min Liu, Qiu-Ni Liang, Er-Jian Wu, Bing-Jie Hu, Xiang-Dong Sun

**Affiliations:** 1 School of Basic Medical Sciences, Key Laboratory of Neurogenetics and Channelopathies of Guangdong Province and the Ministry of Education of China, Institute of Neuroscience and Department of GFNeurology of the Second Affiliated Hospital of Guangzhou Medical University, Guangzhou, China; 2 Department of Neurology of the Sixth Affiliated Hospital, Guangzhou Medical University, Guangzhou, China; 3 Guangzhou Medical University-Guangzhou Institute of Biomedicine and Health (GMU-GIBH) Joint School of Life Sciences, Guangzhou Medical University, Guangzhou, China; 4 Key Laboratory of Mental Health of the Ministry of Education, Guangdong-Hong Kong-Macao Greater Bay Area Center for Brain Science and Brain-Inspired Intelligence, Guangdong-Hong Kong Joint Laboratory for Psychiatric Disorders, Guangdong Province Key Laboratory of Psychiatric Disorders, Guangdong Basic Research Center of Excellence for Integrated Traditional and Western Medicine for Qingzhi Diseases, Department of Neurobiology, School of Basic Medical Sciences, Southern Medical University, Guangzhou, China

**Keywords:** Agrin, Lrp4, Status epilepticus, Epilepsy, Astrocyte, Adenosine

## Abstract

**Background:**

Human patients often experience an episode of serious seizure activity, such as status epilepticus (SE), prior to the onset of temporal lobe epilepsy (TLE), suggesting that SE can trigger the development of epilepsy. Yet, the underlying mechanisms are not fully understood. The low-density lipoprotein receptor related protein (Lrp4), a receptor for proteoglycan-agrin, has been indicated to modulate seizure susceptibility. However, whether agrin-Lrp4 pathway also plays a role in the development of SE-induced TLE is not clear.

**Methods:**

*Lrp4*^*f/f*^ mice were crossed with *hGFAP-Cre* and *Nex-Cre* mice to generate brain conditional Lrp4 knockout mice (*hGFAP-Lrp4*^*−/−*^) and pyramidal neuron specific knockout mice (*Nex-Lrp4*^*−/−*^). Lrp4 was specifically knocked down in hippocampal astrocytes by injecting AAV virus carrying hGFAP-Cre into the hippocampus. The effects of agrin-Lrp4 pathway on the development of SE-induced TLE were evaluated on the chronic seizure model generated by injecting kainic acid (KA) into the amygdala. The spontaneous recurrent seizures (SRS) in mice were video monitored.

**Results:**

We found that *Lrp4* deletion from the brain but not from the pyramidal neurons elevated the seizure threshold and reduced SRS numbers, with no change in the stage or duration of SRS. More importantly, knockdown of Lrp4 in the hippocampal astrocytes after SE induction decreased SRS numbers. In accord, direct injection of agrin into the lateral ventricle of control mice but not mice with *Lrp4* deletion in hippocampal astrocytes also increased the SRS numbers. These results indicate a promoting effect of agrin-Lrp4 signaling in hippocampal astrocytes on the development of SE-induced TLE. Last, we observed that knockdown of Lrp4 in hippocampal astrocytes increased the extracellular adenosine levels in the hippocampus 2 weeks after SE induction. Blockade of adenosine A1 receptor in the hippocampus by DPCPX after SE induction diminished the effects of Lrp4 on the development of SE-induced TLE.

**Conclusion:**

These results demonstrate a promoting role of agrin-Lrp4 signaling in hippocampal astrocytes in the development of SE-induced development of epilepsy through elevating adenosine levels. Targeting agrin-Lrp4 signaling may serve as a potential therapeutic intervention strategy to treat TLE.

## Background

Epilepsy is a severe neurological disease that affects ~ 1% of the population worldwide [1, 2]. Among the various types of epilepsy, temporal lobe epilepsy (TLE) is most prevalent and often devastating due to high rates of drug-resistance and relapse [3]. A common phenomenon in clinic is that patients often experienced an episode of serious seizure activity, such as status epilepticus (SE), prior to the onset of TLE [4, 5], suggesting that the occurrence of SE contributes to the development of TLE. Elucidating the regulatory mechanisms underlying the SE-induced TLE will provide potential targets for prevention and intervention therapies [6].

For a long time, the focus of epilepsy research has been neurocentric, since the primary cellular phenotype of epilepsy is the aberrant synchronized neuronal firings, probably attributed to the imbalance between excitation and inhibition [7]. Nowadays, more and more evidence has suggested an indispensable role of glia, particularly astrocytes, in the pathophysiology of epilepsy [7–9]. For instance, astrogliosis is a hallmark of focal epilepsy [10]. Astrocyte-specific deletion of the β1 integrin gene *Itgb1* causes gliosis and spontaneous recurrent seizures (SRS) [11]. Astrocytes express abundant K^+^ channels such as K_ir_4.1 and water channel-AQP4. A failure to adequately buffer K^+^ and water by astrocytes has been reported to cause neuronal hyperexcitability and seizures [12, 13]. In addition, astrocyte can release various gliotransmitters including glutamate, ATP, and adenosine [14]. Adenosine is a widely accepted endogenous anticonvulsant, whose dysfunction has been found in epilepsies of animals and humans [15]. Nevertheless, the molecular mechanisms underlying adenosine deficits in epilepsy are still not fully understood.

Low-density lipoprotein receptor-related protein 4 (Lrp4) is a type I single transmembrane protein that belongs to the LDL receptor family [16, 17]. Extensive studies have demonstrated that Lrp4, serving as a co-receptor for motor nerve-derived factor-agrin, plays critical roles in the formation, maturation, and maintenance of the neuromuscular junction (NMJ) [18–22]. Lrp4 is also expressed in the brain [23, 24]. In our previous study, we have reported that Lrp4 is enriched in the astrocytes of hippocampal region, where it modulates excitatory presynaptic transmitter release and synaptic plasticity via regulation of astrocytic ATP release [25]. Interestingly, Lrp4 seems vital to seizure threshold as *Lrp4* deletion caused delayed seizure induced by pilocarpine [25], which is further confirmed by a recent paper [26]. These observations suggest that Lrp4 in astrocyte is required for the regulation of seizure susceptibility. However, whether Lrp4 is involved in SE-induced TLE is waiting to be determined.

In the present study, we present in vivo evidence to demonstrate that astrocytic Lrp4 in the hippocampus regulates the development of SE-induced TLE through adenosine signaling, which sheds light on the development of new therapeutic interventions for epilepsy.

## Results

### *Lrp4* deletion in the brain decreases seizure susceptibility and suppresses SE-induced TLE

**Fig. 1:**
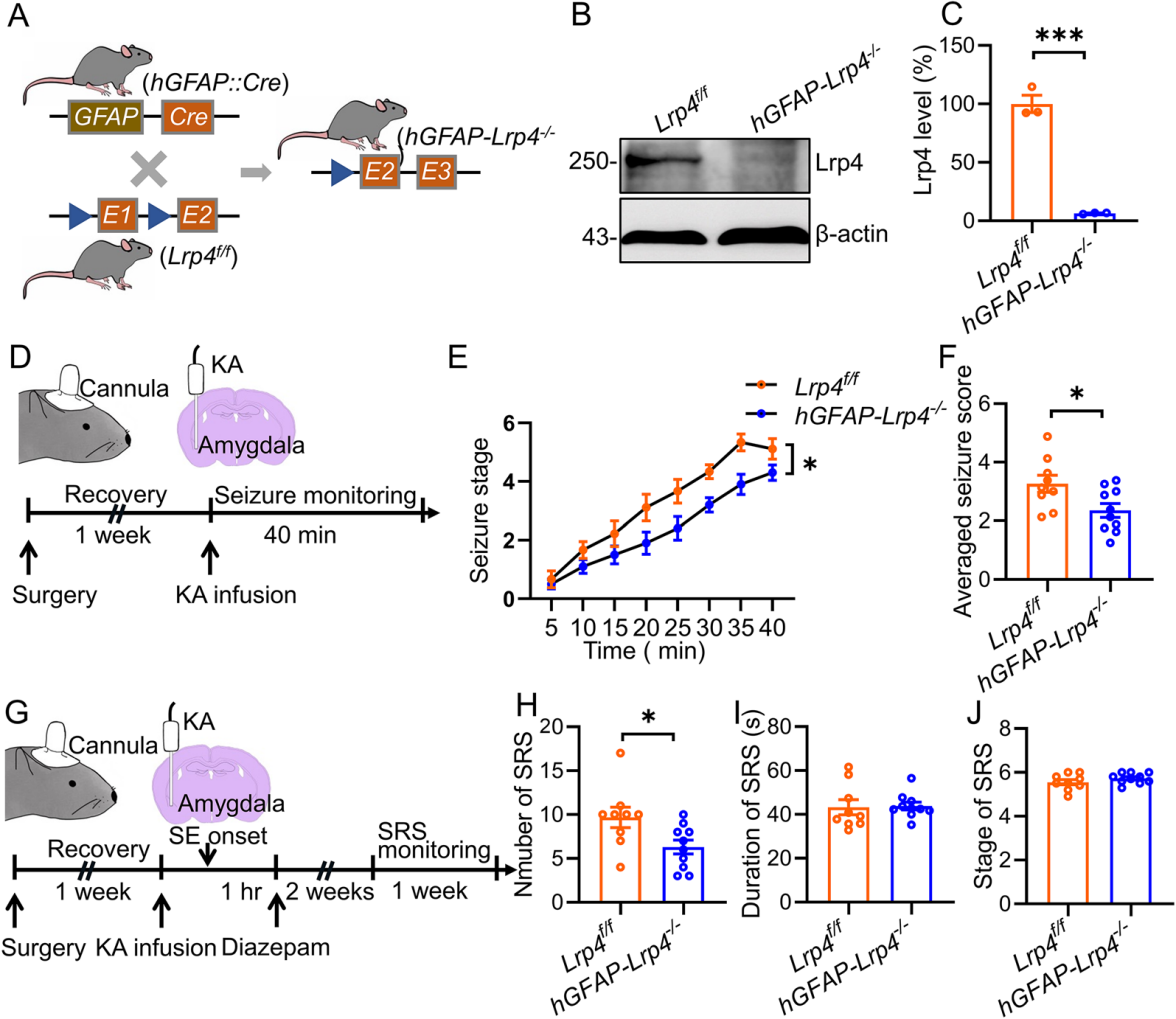
*Lrp4* deletion in the brain decreases seizure susceptibility and suppresses SE-induced TLE. **A** Mice breeding paradigm. *Lrp4*^*f/f*^ mice were crossed with *hGFAP::Cre* mice to eventually generate *hGFAP-Lrp4*^*−/−*^ mice. **B** Reduced Lrp4 expression in the hippocampus of *hGFAP-Lrp4*^*−/−*^ mice. β-actin was taken as a loading control. Shown were representative blots of each genotype. **C** Quantitative analysis of data in **B**. n = 3 mice per group. Student’s t test, t_(4)_ = 12.77, p = 0.0002. **D** Experimental design for seizure induction. One week after cannula implantation into the amygdala, mice were infused with kainic acid (KA) through cannula and seizure behaviors were monitored for 40 min. **E** Decreased seizure stages in *hGFAP-Lrp4*^*−/−*^ mice. n = 9 *Lrp4*^*f/f*^ mice; n = 10 *hGFAP-Lrp4*^*−/−*^ mice. Repeated two-way ANOVA, F_(1,17)_ = 6.127, p = 0.0241. **F** The averaged seizure score was decreased in *hGFAP-Lrp4*^*−/−*^ mice. n = 9 *Lrp4*^*f/f*^ mice; n = 10 *hGFAP-Lrp4*^*−/−*^ mice. Student’s t test, t_(17)_ = 2.475, p = 0.0241. **G** Experimental design for SRS recording. KA was infused into the amygdala to induce SE, which was terminated one hour later by injection of diazepam (i.p). After a latent period of 2 weeks, SRS were recorded for 1 week. **H** Reduced SRS number in *hGFAP-Lrp4*^*−/−*^ mice. n = 9 *Lrp4*^*f/f*^ mice; n = 10 *hGFAP-Lrp4*^*−/−*^ mice. Student’s t test, t_(17)_ = 2.434, p = 0.0263. **I** Simlar duration of SRS between genetypes. n = 9 *Lrp4*^*f/f*^ mice; n = 10 *hGFAP-Lrp4*^*−/−*^ mice. Student’s t test, t_(17)_ = 0.1525, p = 0.8806. **J** Not changed seizure stage in *hGFAP-Lrp4*^*−/−*^ mice. n = 9 *Lrp4*^*f/f*^ mice; n = 10 *hGFAP-Lrp4*^*−/−*^ mice. Student’s t test, t_(17)_ = 1.252, p = 0.2275.

To examine the role of Lrp4 in the development of epilepsy, we generated brain-specific *Lrp4* mutant mice by crossing *hGFAP::Cre* mice with *Lrp4*-flanked (*Lrp4*^*f/f*^) mice (Fig. 1A). It is well established that *hGFAP::Cre* mice express Cre recombinase in the neural progenitor cells, which give rise to neurons and glial cells in the brain [27, 28]. Western blot analysis indicated that the resulting *hGFAP::Cre; Lrp4*^*f/f*^ (*hGFAP-Lrp4*^*−/−*^) mice showed hardly detected Lrp4 protein in the hippocampus (Fig. 1B, C), suggesting a successful *Lrp4* mutation. We utilized a frequently used TLE animal model, in which the KA (0.3 µg in 0.5 µl PBS) was infused into the amygdala to induce seizure (Fig. 1D). The seizure behaviors were continuously monitored after KA infusion. While all mice showed gradually increased seizure scores, indicating a seizure development, *hGFAP-Lrp4*^*−/−*^ mice exhibited significantly lower seizure scores at various time points when compared with *Lrp4*^*f/f*^ mice (Fig. 1E). Accordingly, the average seizure score of *hGFAP-Lrp4*^*−/−*^ mice was lower than that of *Lrp4*^*f/f*^ mice (Fig. 1F). These results suggest that *Lrp4* mutation in the brain decreases seizure susceptibility, in line with previous reports [25, 26]. We then treated mice with diazepam 1 h after SE onset to terminate seizure activity. The SRS was continuously monitored for 1 week after 2 weeks of the latent period (Fig. 1G). We found that the total number of SRS was decreased in *hGFAP-Lrp4*^*−/−*^ mice compared with that in *Lrp4*^*f/f*^ mice, with no change in mean SRS duration or stage (Fig. 1H–J). These observations implicate that Lrp4 deletion in the brain has a suppressive effect on SE-induced TLE.

### Lrp4 in pyramidal neurons is dispensable for seizure susceptibility and SE-induced TLE

**Fig. 2:**
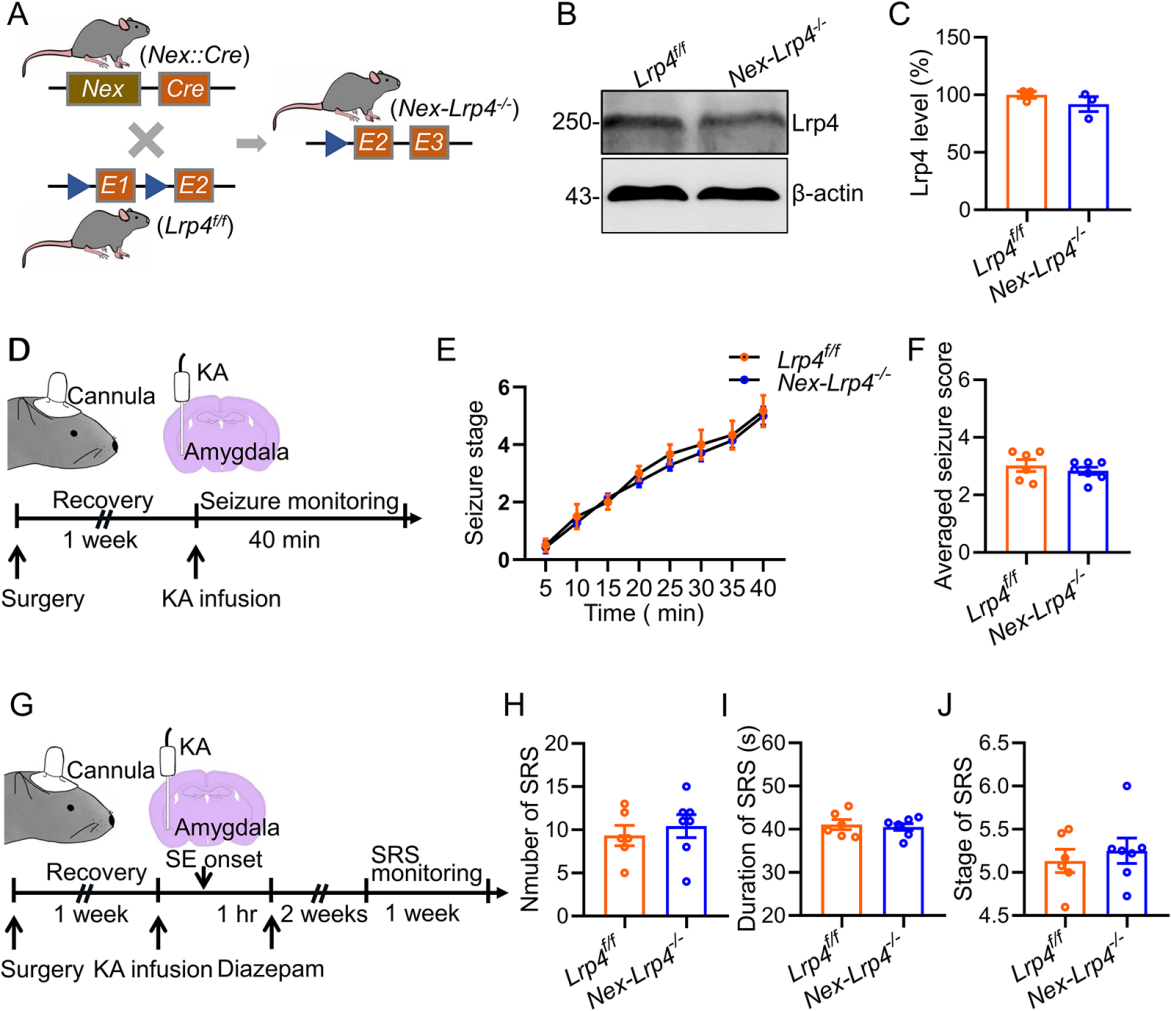
Lrp4 in pyramidal neurons is dispensable for seizure susceptibility and SE-induced TLE. **A** Mice breeding paradigm. *Lrp4*^*f/f*^ mice were crossed with *Nex::Cre* mice to eventually generate *Nex-Lrp4*^*−/−*^ mice. **B** Similar hippocampal Lrp4 level between *Lrp4*^*f/f*^ and *Nex-Lrp4*^*−/−*^ mice. β-actin was taken as a loading control. Shown were representative blots for each genotype. **C** Quantitative analysis of data in **B**. n = 3 mice per group. Student’s t test, t_(4)_ = 1.141, p = 0.3177. **D** Experimental design for seizure induction. One week after cannula implantation into the amygdala, mice were infused with kainic acid (KA) through cannula and seizure behaviors were monitored for 40 min. **E** Not changed seizure stages in *Nex-Lrp4*^*−/−*^ mice. n = 6 *Lrp4*^*f/f*^ mice; n = 7 *Nex-Lrp4*^*−/−*^ mice. Repeated two-way ANOVA, F_(1,11)_ = 0.6033, p = 0.4537. **F** The averaged seizure score was similar between the two genetypes. n = 6 *Lrp4*^*f/f*^ mice; n = 7 *Nex-Lrp4*^*−/−*^ mice. Student’s t test, t_(11)_ = 0.7767, p = 0.4537. **G** Experimental design for SRS recording. KA was infused into the amygdala to induce SE, which was terminated one hour later by injection of diazepam (i.p). After a latent period of 2 weeks, SRS were recorded for 1 week. **H** Not changed SRS number in *Nex-Lrp4*^*−/−*^ mice. n = 6 *Lrp4*^*f/f*^ mice; n = 7 *Nex-Lrp4*^*−/−*^ mice. Student’s t test, t_(11)_ = 0.6087, p = 0.5551. **I** Simlar duration of SRS between the two genetypes. n = 6 *Lrp4*^*f/f*^ mice; n = 7 *Nex-Lrp4*^*−/−*^ mice. Student’s t test, t_(11)_ = 0.4012, p = 0.6959. **J** Not changed seizure stage in *Nex-Lrp4*^*−/−*^ mice. n = 6 *Lrp4*^*f/f*^ mice; n = 7 *Nex-Lrp4*^*−/−*^ mice. Student’s t test, t_(11)_ = 0.5858, p = 0.5699

Previous studies indicated that Lrp4 also expressed in neurons of the developing brain and regulates excitatory synapse formation [29, 30]. To dissect the potential role of neuronal Lrp4 in the development of epilepsy, we generated pyramidal neurons-specific *Lrp4* mutant mice by crossing *Lrp4*^*f/f*^ mice with *Nex::Cre* mice (Fig. 2A), in which Cre expression is under control of Nex promoter and restricted to pyramidal neurons of the dorsal telencephalon [31]. Western blot results showed that Lrp4 level in the hippocampus of *Nex-Lrp4*^*−/−*^ mice was slightly but not significantly decreased (Fig. 2B, C). We used the KA animal model to examine the effects of Lrp4 in pyramidal neurons on seizures susceptibility (Fig. 2D). The seizure development following KA infusion was not different between the two genotypes (Fig. 2E), and so was the averaged seizure score (Fig. 2F). These results suggest a limited role of Lrp4 in pyramidal neurons in seizure susceptibility. We further tested the effect of Lrp4 in pyramidal neurons on the development of SE-induced TLE (Fig. 2G). We found that neither the SRS number nor duration and stage of SRS was changed in *Nex-Lrp4*^*−/−*^ mice when compared to *Lrp4*^*f/f*^ mice (Fig. 2H–J). Collectively, these data suggest that Lrp4 in pyramidal neurons is dispensable for seizure susceptibility and SE-induced epilepsy.

### Deletion of astrocytic Lrp4 in the hippocampus suppresses SE-induced TLE

**Fig. 3:**
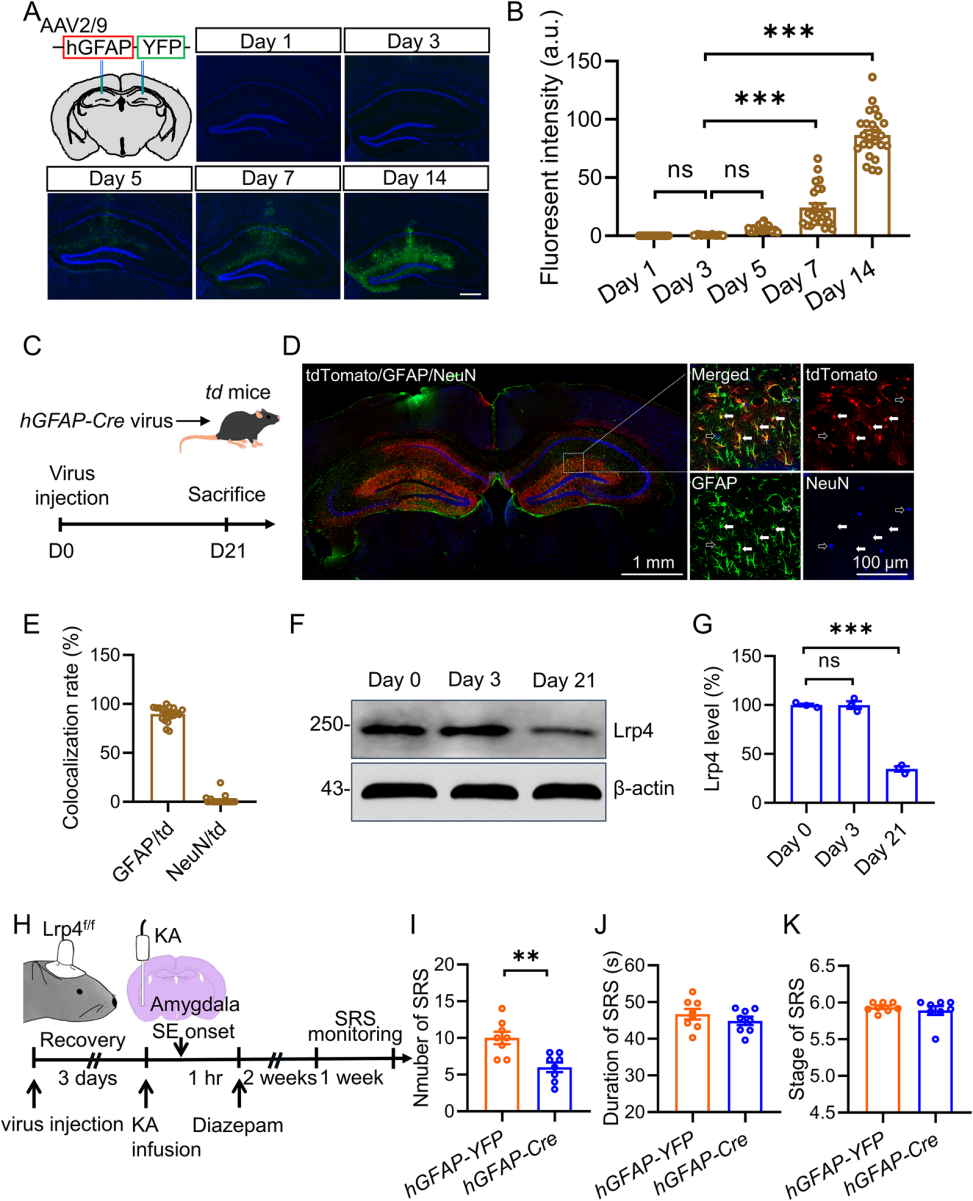
Deletion of astrocytic Lrp4 in the hippocampus suppresses SE-induced TLE. **A** Representative images of YFP expression in *hGFAP-YFP* virus-injected mice. Hippocampal sections were collected at various time points after stereotaxic microinjection of AAV2/9-*hGFAP*-Cre virus. Scale bar: 500 µm. **B** Gradually increased YFP expression with time. n = 17 hippocampi from 3 mice for Day 1 and Day 3; n = 22 hippocampi from 3 mice for Day 5; n = 24 hippocampi from 3 mice for Day 14. One-way ANOVA, F_(4,99)_ = 187.2, p < 0.0001. Tukey’s post hoc test, Day 1 vs Day 3, p > 0.9999; Day 3 vs Day 5, p = 0.7227; Day 3 vs Day 7, p < 0.0001; Day 3 vs Day 14, p < 0.0001. **C** Experimental design. *hGFAP-Cre* virus was bilaterally injected into the hippocampus of td mice. 21 days later, mice were sacrificed and subjected to immunostaining. **D** Representative images of hippocampal slices from *hGFAP-Cre* virus injected mice co-stained with and astrocyte marker-GFAP (Green) and neuronal marker-NeuN (Blue). Scale bar: 1 mm (Left) and 100 µm (Right). **E** Quantification of colocalization rates. **F** Decreased hippocampal Lrp4 level 21 days after *hGFAP-Cre* virus was injected into the hippocampus of *Lrp4*^*f/f*^ mice. β-actin was taken as a loading control. Shown were representative blots for each group. **G** Quantitative analysis of data in **F**. n = 3 mice per group. One-way ANOVA, F_(2,6)_ = 177.5, p < 0.0001. Tukey’s post hoc test, Day 1 vs Day 3, p > 0.9999; Day 0 vs Day 21, p < 0.0001. **H** Experimental design. Virus was bilaterally injected into the hippocampus of *Lrp4*^*f/f*^ mice and a guide canula was implanted in the right amygdala. 3 days later, KA was infused into the amygdala to induce SE, which was terminated one hour later by injection of diazepam (i.p). After a latent period of 2 weeks, SRS were recorded for 1 week. **I** Decreased SRS number in *hGFAP-Cre* group. n = 8 mice for each group. Student’s t test, t_(14)_ = 3.742, p = 0.0022. **J** Simlar duration of SRS between the two groups. n = 8 mice for each group. Student’s t test, t_(14)_ = 0.9821, p = 0.3427. **K** Not changed seizure stage in *hGFAP-Cre* group. n = 8 mice for each group. Student’s t test, t_(14)_ = 0.6704, p = 0.5135

Our previous study indicated that Lrp4 expressed mainly in the astrocyte of hippocampus and functions as an excitatory neurotransmission modulator [25]. To specifically investigate whether astrocytic Lrp4 in the hippocampus plays a role in the development of SE-induced TLE, we attempted to knock down *Lrp4* in the astrocytes of hippocampus after SE onset by bilateral injection of adeno-associated virus (AAV) vectors expressing *hGFAP-Cre* into the hippocampus. To find an appropriate time point for KA infusion in order to avoid *Lrp4* knockdown before SE onset, we determined when the gene of interest starts to express after virus injection. While the GFP expression was barely detectable at day one and day three after *hGFAP-YFP* virus injection, a slight YFP expression was found on day 5 and gradually increased with time (Fig. 3A, B). This result implicates that day three after virus injection is an applicable timepoint for KA infusion. We also verified the specificity of *hGFAP-Cre* virus by injecting it into the hippocampus of Rosa:: LSL-tdTomato (td) reporter mice (Fig. 3C). Quantitative analysis revealed that most of td + cells (~ 90%) co-localized with astrocyte marker-GFAP, although there is a low co-localization rate between td and neuron marker-NeuN (~ 2%) (Fig. 3D, E). Furthermore, we examined the Lrp4 expression in the hippocampus at different timepoint after *hGFAP-Cre* virus injection into hippocampus of *Lrp4*^*f/f*^ mice. Western blot results showed that Lrp4 level at day 3 was comparable to that at day 0, whereas Lrp4 level at day 21 was dramatically decreased compared with that at day 0 (Fig. 3F, G), being consistent with the results of the YFP expression pattern (Fig. 3B). We then tested the effect of Lrp4 in the astrocytes on the development of SE-induced TLE (Fig. 3I). We found that the SRS number was significantly decreased in *hGFAP-Cre* virus injected *Lrp4*^*f/f*^ mice (refer to *hGFAP-Cre* thereafter) compared with that in *hGFAP-YFP* virus injected *Lrp4*^*f/f*^ mice (refer to *hGFAP-YFP* thereafter) (Fig. 3J). In contrast, the duration and stage of SRS were not different between the two groups (Fig. 3K, H). Altogether, these data suggest that deletion of astrocytic Lrp4 in the hippocampus exhibits suppressive effects on SE-induced epilepsy.

### Agrin promotes the development of SE-induced TLE in an Lrp4-dependent manner

**Fig. 4:**
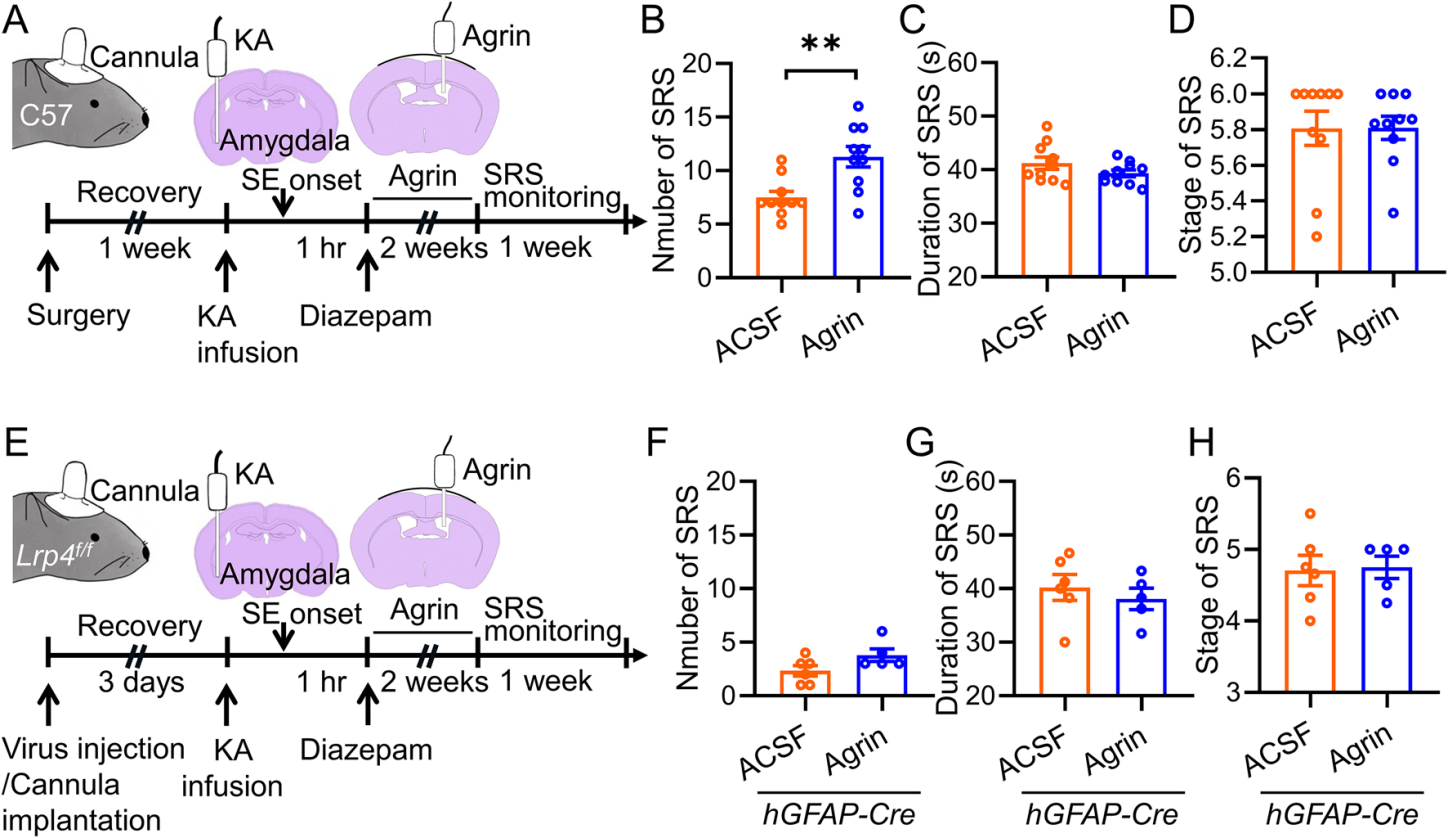
Agrin promotes the development of SE-induced TLE in an Lrp4-dependent manner. **A** Experimental design for SRS recording. KA was infused into the amygdala of C57 wildtype mice to induce SE, which was terminated one hour later by injection of diazepam (i.p). During the latent period of 2 weeks, agrin was infused into the lateral ventricle each day. SRS were then recorded for 1 week. **B** Increased SRS number in Agrin-treatment group. n = 10 mice for each group. Student’s t test, t_(18)_ = 3.428, p = 0.003. **C** Simlar duration of SRS between the two groups. n = 10 mice for each group. Student’s t test, t_(18)_ = 0.429, p = 0.1701. **D** Simlar seizure stage between the two groups. n = 10 mice for each group. Student’s t test, t_(18)_ = 0.021, p = 0.9835. **E** Experimental design. Virus was bilaterally injected into the hippocampus of *Lrp4*^*f/f*^ mice and a guide canula was implanted in the right amygdala. 3 days later, KA was infused into the amygdala to induce SE, which was terminated one hour later by injection of diazepam (i.p). During the latent period of 2 weeks, agrin was infused into the lateral ventricle each day. SRS were then recorded for 1 week. **F** Diminished change in SRS number in *hGFAP-Cre* group by Agrin-treatment. n = 6 mice for ACSF group; n = 5 mice for agirn group. Student’s t test, t_(9)_ = 1.933, p = 0.0853. **G** Simlar duration of SRS between the two groups. n = 6 mice for ACSF group; n = 5 mice for agirn group. Student’s t test, t_(9)_ = 0.6621, p = 0.5245. **H** Simlar SRS stage between the two groups. n = 6 mice for ACSF group; n = 5 mice for agirn group. Student’s t test, t_(9)_ = 0.1574, p = 0.8784

It is known that agrin is a ligand of Lrp4 in the NMJ region that promotes NMJ formation and maintenance [32]. Agrin is also expressed in the brain [33]. To examine the role of agrin in SE-induced epilepsy, we injected exogenous agrin protein (1 µl, 50 µg/ml) into the lateral ventricle each day for 2 weeks after SE onset (Fig. 4A). We found that the SRS number in agrin-injected mice was dramatically increased compared with that in artificial cerebrospinal fluid (ACSF)-injected mice (Fig. 4B). In contrast, the duration and stage of SRS were not different (Fig. 4C, D). These results suggest a promotive effect of agrin on SE-induced epilepsy. To detect whether the effect of agrin on the development of SE-induced TLE relies on astrocytic Lrp4 in the hippocampus, we knocked down Lrp4 in the astrocytes by injecting *hGFAP-Cre* virus into the hippocampus of *Lrp4*^*f/f*^ mice before SE induction. We found that the number, duration, and stage of SRS were all comparable between agrin and ACSF treated groups (Fig. 4E-H). Together, these observations suggest that agrin promotes SE-induced epilepsy in an Lrp4-dependent manner.

### Adenosine signaling is required for Lrp4 to regulate the development of SE-induced TLE

**Fig. 5:**
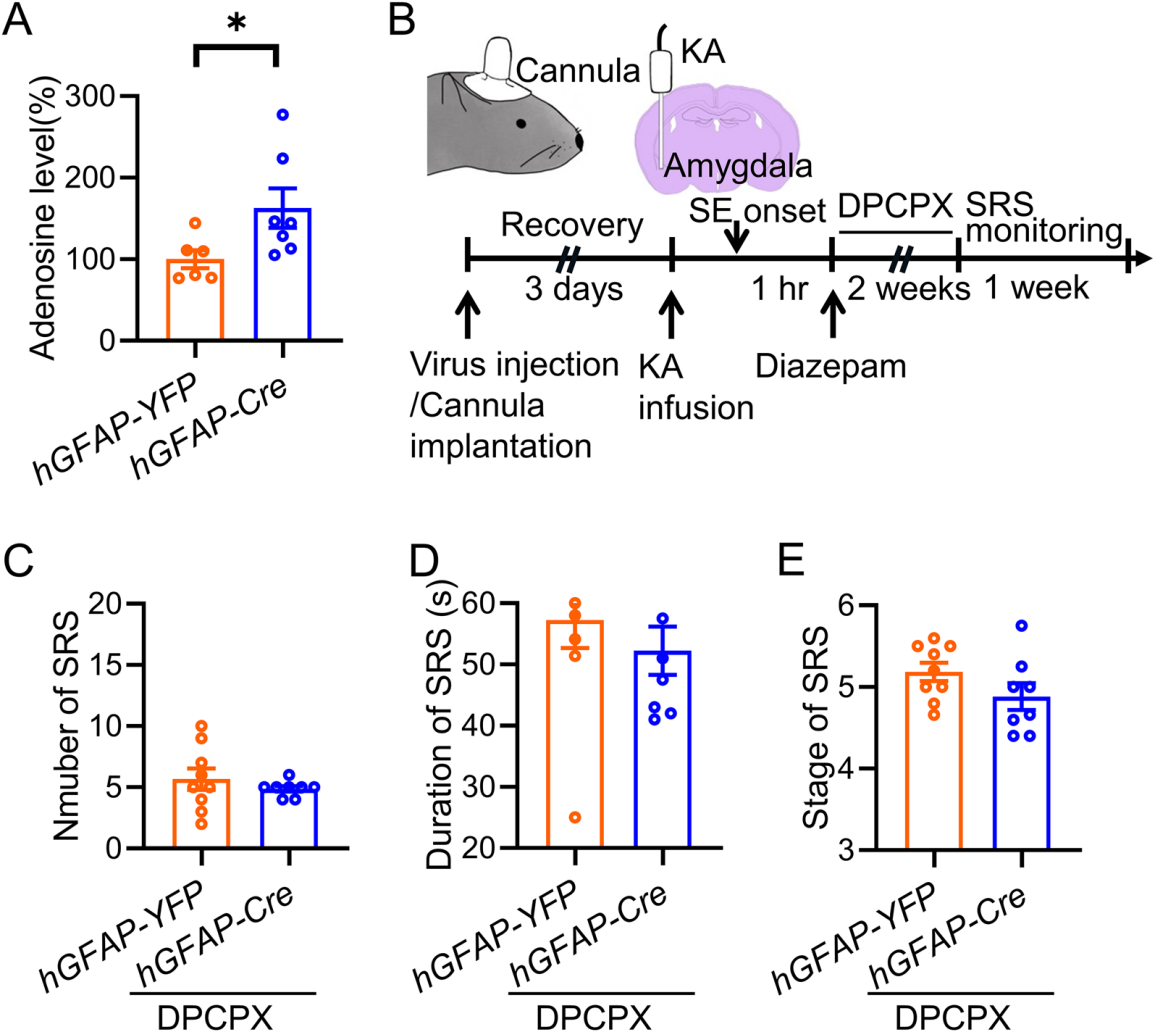
Adenosine signaling is required for Lrp4 to regulate the development of SE-induced TLE. **A** Increased adenosine level in the hippocampus of *hGFAP-Cre* virus injected mice. n = 6 mice for *hGFAP-YFP* group; n = 7 mice for *hGFAP-Cre* group. Student’s t test, t_(11)_ = 2.239, p = 0.0468. **B** Experimental design for SRS recording. A virus was bilaterally injected into the hippocampus of *Lrp4*^*f/f*^ mice and a guide canula was implanted in the right amygdala. Three days later, KA was infused into the amygdala to induce SE, which was terminated one hour later by injection of diazepam (i.p). During the latent period of 2 weeks, DPCPX was i.p injected each day. SRS were then recorded for 1 week. **C** Diminished change in SRS number in *hGFAP-Cre* group by DPCPX treatment. n = 9 mice for *hGFAP-YFP* group; n = 8 mice for *hGFAP-Cre* group. Student’s t test, t_(15)_ = 0.8224, p = 0.4237. **D** Simlar duration of SRS between the two groups. n = 9 mice for *hGFAP-YFP* group; n = 8 mice for *hGFAP-Cre* group. Student’s t test, t_(15)_ = 0.8227, p = 0.4236. **E** Simlar SRS stage between the two groups. n = 9 mice for *hGFAP-YFP* group; n = 8 mice for *hGFAP-Cre* group. Student’s t test, t_(15)_ = 1.547, p = 0.1427

Adenosine is considered as an endogenous anti-convulsant [15]. In our previous work we found that deletion of Lrp4 in the astrocyte increased ATP release, which converted quickly to adenosine and promote presynaptic glutamate release through A1R receptor. To investigate the downstream mechanism through which Lrp4 regulates the development of SE-induced TLE, we measured the extracellular adenosine level in the hippocampus. In line with the previous report, the extracellular adenosine level was dramatically increased after Lrp4 knockdown in the hippocampal astrocytes (Fig. 5A). We then injected the antagonist of adenosine-DPCPX (1 mg/kg, i.p) after SE onset each day for 2 weeks (Fig. 5B). Remarkably, neither the number nor the duration and stage of SRS was changed between *hGFAP-YFP* and *hGFAP-Cre* groups (Fig. 5C-E), indicating that adenosine signaling is required for Lrp4 in regulation of the development of SE-induced TLE.

## Discussion

Our study identifies a vital role of agrin-Lrp4 signaling in regulating the development of SE-induced epilepsy. First, deletion of *Lrp4* in the brain caused decreased seizure susceptibility and suppressed the development of SE-induced TLE. However, specific knockout of Lrp4 in the pyramidal neurons failed to show these phenotypes. Remarkably, a particular knockdown of astrocytic Lrp4 in the hippocampus is sufficient to duplicate the phenotypes of *Lrp4* brain mutant mice. Second, we demonstrated that agrin exhibited a promotive effect on the development of SE-induced TLE and this effect rely on astrocytic Lrp4 in the hippocampus. Last, knockdown of Lrp4 in astrocyte elevated the extracellular adenosine level in the hippocampus and blockade of adenosine signaling diminished the effect of Lrp4 on the development of SE-induced TLE. Together, our results indicate that agrin-Lrp4 signaling affects the development of SE-induced TLE via adenosine signaling, which will shed light on the novel therapeutic intervention strategy to treat TLE.

Epilepsy is one of the toughest pathological conditions that no effective pharmacological interventions so far were able to prevent its occurrence, which presses the urge to elucidate its pathological mechanisms and identify novel therapeutic targets [34]. It has been reported that during the development of epilepsy following an initial brain insults, such as SE, neuronal excitation imbalance, network reorganization, gliosis and even neuronal death occur chronically in the brain, especially in the temporal lobe region [3]. It is nowadays increasingly recognized that astrocytic dysfunctions contribute tremendously to the development of epilepsy [9]. Under conditions of epilepsy, astrocytes undergo extensive changes including Ca^2+^ signaling exacerbation [35], gap junction uncoupling [36, 37], energy metabolism [9], ion and neurotransmitter homeostasis [38], all of which could lead to neuronal hyperexcitability, accordingly giving rising to and/or aggravating epilepsy pathogenesis. In the present study, we identify a new astrocyte-originated molecular mechanism underlying the regulation of the development of SE-induced TLE. By using in vivo gene manipulation strategy, we demonstrate that Lrp4 in astrocyte of the hippocampus, probably in response to its ligand-agrin, promotes SE-induced TLE. Nevertheless, whether the effects of Lrp4 signaling on epilepsy is generally applied in distinct epilepsy models, such as pilocarpine and pentylenetetrazol-induced seizure models, is not clear, which is worth being examined in future study.

The roles of agrin-Lrp4 signaling in the brain has been continuously uncovered [39]. Results from various groups have shown that Lrp4 is expressed in cultured embryonic cortical neurons [40], neurons in the developing central nervous system [29], astrocytes [25], and neuronal stem/progenitor cells in the adult brain [41]. Whether Lrp4 is expressed in the neurons of adult brain is controversial. Although Lrp4 was shown to localize to the nerve terminals in the adult Drosophila brain [30], deletion of *Lrp4* gene in the pyramidal neurons of mice (*Nex-Lrp4*^*−/−*^) caused little effect on Lrp4 expression in the hippocampus, as reported in a previous study and the present study [25]. These results suggest a minor (if any) expression of Lrp4 in the excitatory neurons in the adult brain. Besides, we found that *Nex-Lrp4*^*−/−*^ mice exhibited similar seizure threshold and SE-induced TLE to *Lrp4*^*f/f*^ mice, providing further evidence for dispensable role of Lrp4 in the pyramidal neurons. In contrast, we report that specific knockdown of *Lrp4* in the astrocytes of the hippocampus duplicated the phenotypes of brain-wide *Lrp4* mutant mice (*hGFAP-Lrp4*^*−/−*^), demonstrating that astrocytic Lrp4 in the hippocampus is critical in regulation of the development of SE-induced epilepsy. Please note that in the present study we only studied the role of astrocytic Lrp4 in the hippocampus on status epilepticus-induced epilepsy at adult stage. In light that epilepsy could occur at any age and the incidence rate is higher in children than in adult [42, 43], it would be interesting to examine whether the effects of Lrp4 in hippocampal astrocytes on epilepsy varies at different age in the future study. On the other hand, it is still not clear whether Lrp4 locates in the GABAergic inhibitory neurons in the brain and plays a role in the development of epilepsy, which warrants further investigation. Agrin is considered a modulator of synaptogenesis that expressed in the excitatory neurons in the hippocampus and upregulated following acute seizure induction in adult rat brain [41, 44]. Agrin heterozygous mice showed decreased seizures stage and mortality [45]. We found that agrin infusion into ventricle promoted the development of SE-induced epilepsy which is dependent of Lrp4 in the astrocytes. A parsimonious interpretation is that SE induces over-release of agrin in the hippocampus, which binds to astrocytic Lrp4 and aggregates the development of SE-induce epilepsy.

It is well established that adenosine is an endogenous anti-convulsant and seizure terminator in the brain [46]. Acute seizure increased adenosine level transiently, which may serve as a self-terminating mechanism [47].However, the adenosine level becomes low during late phase of epileptogenesis [15]. One proposed reason is that adenosine kinase (ADK), which is predominantly expressed in the astrocytes [48], is overexpressed because of astrogliosis [10]. We found that deletion of Lrp4 in the astrocytes increased extracellular adenosine level during the development of epilepsy, providing a novel molecular mechanism for adenosine regulation. How is adenosine level regulated by Lrp4 during epilepsy is not understood yet. Results from a previous study have indicated that Lrp4 in astrocyte regulates ATP release, which could be degraded to adenosine quickly in the extracellular space [25]. Adenosine is also reported to be able to be directly released from the astrocytes [49]. The exact mechanisms underlying Lrp4 regulation of adenosine during the development of epilepsy await to be determined in future study. Besides, adenosine functions as anticonvulsant through binding to multiple receptors, in which A_1_R and A_2A_R have received most attentions. Mounting evidences have demonstrated that A_1_R exerts an inhibitory effect on excitatory synaptic activity, whereas A_2A_R is stimulatory in response to adenosine activation [50]. In the present study, we found that treatment of A_1_R antagonist DPCPX diminished the effects of *Lrp4* deletion on the development of SE-induced TLE, suggesting that A_1_R is the main downstream target for enhanced adenosine level caused by *Lrp4* deletion. Note that we are unable to exclude the possible involvement of other adenosine receptors in the process of Lrp4 regulation of the development of SE-induced TLE. More direct evidence is warranted in future study to support the conclusion.

## Materials and methods

### Reagents and antibodies

Chemicals were purchased from Sigma-Aldrich unless otherwise indicated. The following primary antibodies were used: mouse anti-Lrp4 (BioLegend) (832201; 1:1000 for blotting); rabbit anti-GFAP (Cell Signaling Technology) (80788 s; 1:100 for staining); mouse anti-NeuN (Millipore) (MAB377, 1:100 for staining).

### Animals

Adult male mice (Eight to twelve weeks) were used in the experiments. The detailed information about mouse strains, including *Lrp4*^*f/f*^, *hGFAP::Cre* and *Nex::Cre* has been described previously [25, 51]. Rosa::LSL-tdTomato were purchased from Jackson Labs (#007909). Genotyping procedures were as follows: *Lrp4*^*f/f*^, 5′-CTCTC CCAGC TAAGT CCAGG A-3′ and 5′-CCTCC ATACT GTCTG TGAAT G-3′; *hGFAP::Cre*, 5′-ACT CCT TCA TAA AGC CCT-3′ and 5′-GCC AGC TAC GTT GCT CAC TA-3′; *Nex::Cre*, 5′-GAG TCC TGG AAT CAG TCT TTT TC-3′, 5′-ATC ACT CGT TGC ATC GAC CG-3′ and 5′-CCG CAT AAC CAG TGA AAC AG-3′. In all experiments, significant efforts are made to minimize the total number of animals used while maintaining statistically valid group numbers. Mice were housed in a condition with a temperature of 22 ± 1 °C, > 30% humidity and a standard 12 h light/dark cycle. All animal experimental protocols were approved by the Animal Ethics Committee of Guangzhou Medical University.

### Western blotting

Western blotting was performed as described in our previous study [52]. In brief, brain tissue was homogenized in a RIPA Buffer containing (in mM): 50 Tris–HCl, pH 7.4, 150 NaCl, 2 EDTA, 1 PMSF, 50 sodium fluoride, 1 sodium vanadate, 1 DTT with 1% sodium deoxycholate, 1% SDS and 1% protease inhibitors cocktails. Samples were resolved on SDS/PAGE, transferred to nitrocellulose membranes, and blocked in TBS buffer containing 0.1% Tween-20 and 5% milk for 1 h at room temperature prior to incubation with primary antibodies (overnight at 4 °C). After washing, the membranes were incubated with HRP-conjugated secondary antibodies (Absin ImmunoResearch) in TBS buffer for 1 h at room temperature. Immunoreactive complex bands were visualized using enhanced chemiluminescence (Pierce) and captured using the Genesys imaging system (Gene Company Limited, UK). Band densities of interested proteins were normalized with loading control.

### Cannula implantation and drug infusion

As described previously [53], mice were anesthetized with isoflurane (2–3%) and head-fixed in a stereotaxic device (RWD Life Science.Inc). An incision was made in the scalp and a small hole was drilled into the skull. The guide cannula (IO: 0.48 mm; RWD Life Science.Inc) was implanted inside the right amygdala (coordinates: anteroposterior, − 1.22 mm; dorsoventral, − 4.5 mm; mediolateral, 3 mm relative to bregma) or the left lateral ventricle (coordinates: anteroposterior, − 0.46 mm; dorsoventral, − 2.25 mm; mediolateral, − 1.25 mm relative to bregma), and cemented onto the skull with dental cement. Mice were recovered in their homecages for at least 1 week.

In some experiments, after mice were gently restrained, human recombinant agrin protein (50 ng/mouse, R&D systems, 6624-AG-050) was infused through the infusion cannula (IO: 0.3 mm; RWD Life Science.Inc) into the lateral ventricle at a rate of 20 nl/s, controlled by a microinjector (NanojectIII, Drummond Scientific).

### Seizure induction and behavioral monitoring

As described previously [52], an infusion cannula (IO: 0.3 mm; RWD Life Science.Inc) was inserted into the amygdala through the guide cannula. 0.15 µl of KA (3 mg/ml, Sigma, #420318) was infused at a flow rate of 2 nl/s controlled by microinjector (NanojectIII, Drummond Scientific). The cannula was kept for an additional two mins after completion of infusion and withdrew slowly to minimize reflux along the injection tract. Seizure stages were classified according to the criteria described by Racine [54] and scored every 5 min by a blinded investigator: stage 0, no seizure; stage 1, arrest and rigid posture; stage 2, head nodding; stage 3, sporadic full-body shaking, spasms; stage 4, chronic full-body spasms; stage 5, jumping, shrieking, falling over; stage 6, violent convulsions or death. Seizures at stage 4–6 that last for ≥ 30 min was defined as SE.

To monitor SE-induced SRSs, diazepam (8 mg/kg, i.p.) was injected 1 h after SE induction to terminate seizures. After a latent period of 2 weeks, mice were video monitored from 8 am to 8 pm each day for 1 week. In some experiments, DPCPX (1 mg/kg, Sigma, #C101) was i.p. injected each day during latent period. SRSs, defined as seizures with score ≥ 4, were counted by a blinded investigator.

### Virus injection

Virus injection was performed as described previously [52]. Briefly, after mice were anesthetized with isoflurane (2–3%) and head-fixed in a stereotaxic device (RWD Life Science. Inc), an incision was made in the scalp and four small holes, two on each side, drilled into the skull. Viruses at a volume of 200 µl were injected bilaterally into the dorsal and ventral hippocampus, respectively (coordinates of dorsal hippocampus from bregma: AP, − 1.5 mm; DV, − 2 mm; ML, ± 1.25 mm; coordinates of ventral hippocampus from bregma: AP, − 2.54 mm; DV, − 2.5 mm; ML, ± 2.75 mm) through a pulled glass capillary, controlled by the same microinjector (NanojectIII, Drummond Scientific) at a slow rate of 10 nl/min. The capillary was slowly retracted 10 min after injection. Mice were recovered in their homecages for 3 weeks and subjected to the following experiments. Used recombinant adeno-associated viral (AAV) vectors were purchased from BrainVTA, containing: *AAV2/9-hGFAP-Cre*, titre: 2.21 × 10^12^ v.g./ml, dilution: 1:400, 0.2 μl/injection; *AAV2/9-hGFAP-YFP*, titre: 5.74 × 10^12^ v.g./ml, dilution: 1:400, 0.2 μl/injection.

### Adenosine test

Adenosine test was performed as described previously [25]. In brief, adenosine is measured with the Adenosine Assay Kit (K327-100, BioVision). Samples were incubated with adenosine deminase inhibitor EHNA hydrochloride (E114, Sigma) to inhibit adenosine degradation. Fluorescence was measured using a microplate reader (TECAN, Infinite 200 PRO). Adenosine in samples was calculated based on a calibration curve from standard adenosine samples.

### Statistical analysis

Statistical analyses were performed using GraphPad Prism (GraphPad Software). Sample size choice was made based on previous studies [25, 55]. Student’s *t*-test and one-way ANOVA with Tukey’s post hoc test were used to compare data from two groups and more than two groups, respectively. Repeated two-way ANOVA was used for seizure development studies. All tests were two-sided. Data represent mean ± SEM. p < 0.05 was considered to be statistically significant.

## Data Availability

The data and materials supporting the current study are available from the corresponding author upon reasonable request.

## References

[1] Thijs RD, Surges R, O'Brien TJ, Sander JW (2019). Epilepsy in adults. Lancet.

[2] Moshé SL, Perucca E, Ryvlin P, Tomson T (2015). Epilepsy: new advances. Lancet.

[3] Tatum WO (2012). Mesial temporal lobe epilepsy. J Clin Neurophysiol.

[4] French JA, Williamson PD, Thadani VM, Darcey TM, Mattson RH, Spencer SS, Spencer DD (1993). Characteristics of medial temporal lobe epilepsy: I. Results of history and physical examination. Ann Neurol.

[5] Liu G, Gu B, He XP, Joshi RB, Wackerle HD, Rodriguiz RM, Wetsel WC, McNamara JO (2013). Transient inhibition of TrkB kinase after status epilepticus prevents development of temporal lobe epilepsy. Neuron.

[6] Pitkänen A (2010). Therapeutic approaches to epileptogenesis–hope on the horizon. Epilepsia.

[7] Patel DC, Tewari BP, Chaunsali L, Sontheimer H (2019). Neuron-glia interactions in the pathophysiology of epilepsy. Nat Rev Neurosci.

[8] Nikolic L, Nobili P, Shen W, Audinat E (2020). Role of astrocyte purinergic signaling in epilepsy. Glia.

[9] Boison D, Steinhäuser C (2018). Epilepsy and astrocyte energy metabolism. Glia.

[10] Sofroniew MV (2014). Astrogliosis. Cold Spring Harb Perspect Biol.

[11] Ortinski PI, Dong J, Mungenast A, Yue C, Takano H, Watson DJ, Haydon PG, Coulter DA (2010). Selective induction of astrocytic gliosis generates deficits in neuronal inhibition. Nat Neurosci.

[12] Djukic B, Casper KB, Philpot BD, Chin LS, McCarthy KD (2007). Conditional knock-out of Kir4.1 leads to glial membrane depolarization, inhibition of potassium and glutamate uptake, and enhanced short-term synaptic potentiation. J Neurosci.

[13] Binder DK, Yao X, Zador Z, Sick TJ, Verkman AS, Manley GT (2006). Increased seizure duration and slowed potassium kinetics in mice lacking aquaporin-4 water channels. Glia.

[14] Harada K, Kamiya T, Tsuboi T (2015). Gliotransmitter release from astrocytes: functional, developmental, and pathological implications in the brain. Front Neurosci.

[15] Boison D (2016). Adenosinergic signaling in epilepsy. Neuropharmacology.

[16] Herz J, Strickland DK (2001). LRP: a multifunctional scavenger and signaling receptor. J Clin Invest.

[17] Shen C, Xiong WC, Mei L (2015). LRP4 in neuromuscular junction and bone development and diseases. Bone.

[18] Zhang B, Luo S, Wang Q, Suzuki T, Xiong WC, Mei L (2008). LRP4 serves as a coreceptor of agrin. Neuron.

[19] Weatherbee SD, Anderson KV, Niswander LA (2006). LDL-receptor-related protein 4 is crucial for formation of the neuromuscular junction. Development.

[20] Barik A, Lu Y, Sathyamurthy A, Bowman A, Shen C, Li L, Xiong WC, Mei L (2014). LRP4 is critical for neuromuscular junction maintenance. J Neurosci.

[21] Wu H, Lu Y, Shen C, Patel N, Gan L, Xiong WC, Mei L (2012). Distinct roles of muscle and motoneuron LRP4 in neuromuscular junction formation. Neuron.

[22] Kim N, Stiegler AL, Cameron TO, Hallock PT, Gomez AM, Huang JH, Hubbard SR, Dustin ML, Burden SJ (2008). Lrp4 is a receptor for Agrin and forms a complex with MuSK. Cell.

[23] Tian QB, Suzuki T, Yamauchi T, Sakagami H, Yoshimura Y, Miyazawa S, Nakayama K, Saitoh F, Zhang JP, Lu Y, Kondo H, Endo S (2006). Interaction of LDL receptor-related protein 4 (LRP4) with postsynaptic scaffold proteins via its C-terminal PDZ domain-binding motif, and its regulation by Ca/calmodulin-dependent protein kinase II. Eur J Neurosci.

[24] Gomez AM, Froemke RC, Burden SJ (2014). Synaptic plasticity and cognitive function are disrupted in the absence of Lrp4. Elife.

[25] Sun XD, Li L, Liu F, Huang ZH, Bean JC, Jiao HF, Barik A, Kim SM, Wu H, Shen C, Tian Y, Lin TW, Bates R, Sathyamurthy A, Chen YJ, Yin DM, Xiong L, Lin HP, Hu JX, Li BM, Gao TM, Xiong WC, Mei L (2016). Lrp4 in astrocytes modulates glutamatergic transmission. Nat Neurosci.

[26] Yu Z, Zhang M, Luo B, Jing H, Yu Y, Wang S, Luo S (2020). Lrp4 in hippocampal astrocytes serves as a negative feedback factor in seizures. Cell Biosci.

[27] Dong Z, Chen W, Chen C, Wang H, Cui W, Tan Z, Robinson H, Gao N, Luo B, Zhang L, Zhao K, Xiong WC, Mei L (2020). CUL3 deficiency causes social deficits and anxiety-like behaviors by impairing excitation-inhibition balance through the promotion of cap-dependent translation. Neuron.

[28] Zhuo L, Theis M, Alvarez-Maya I, Brenner M, Willecke K, Messing A (2001). hGFAP-cre transgenic mice for manipulation of glial and neuronal function in vivo. Genesis.

[29] Karakatsani A, Marichal N, Urban S, Kalamakis G, Ghanem A, Schick A, Zhang Y, Conzelmann KK, Rüegg MA, Berninger B, de Ruiz Almodovar C, Gascón S, Kröger S (2017). Neuronal LRP4 regulates synapse formation in the developing CNS. Development.

[30] Mosca TJ, Luginbuhl DJ, Wang IE, Luo L (2017). Presynaptic LRP4 promotes synapse number and function of excitatory CNS neurons. Elife.

[31] Goebbels S, Bormuth I, Bode U, Hermanson O, Schwab MH, Nave KA (2006). Genetic targeting of principal neurons in neocortex and hippocampus of NEX-Cre mice. Genesis.

[32] Li L, Xiong WC, Mei L (2018). Neuromuscular junction formation, aging, and disorders. Annu Rev Physiol.

[33] Ksiazek I, Burkhardt C, Lin S, Seddik R, Maj M, Bezakova G, Jucker M, Arber S, Caroni P, Sanes JR, Bettler B, Ruegg MA (2007). Synapse loss in cortex of agrin-deficient mice after genetic rescue of perinatal death. J Neurosci.

[34] Pitkänen A, Lukasiuk K, Dudek FE, Staley KJ (2015). Epileptogenesis. Cold Spring Harb Perspect Med.

[35] Sano F, Shigetomi E, Shinozaki Y, Tsuzukiyama H, Saito K, Mikoshiba K, Horiuchi H, Cheung DL, Nabekura J, Sugita K, Aihara M, Koizumi S (2021). Reactive astrocyte-driven epileptogenesis is induced by microglia initially activated following status epilepticus. JCI Insight.

[36] Bedner P, Dupper A, Hüttmann K, Müller J, Herde MK, Dublin P, Deshpande T, Schramm J, Häussler U, Haas CA, Henneberger C, Theis M, Steinhäuser C (2015). Astrocyte uncoupling as a cause of human temporal lobe epilepsy. Brain.

[37] Onodera M, Meyer J, Furukawa K, Hiraoka Y, Aida T, Tanaka K, Tanaka KF, Rose CR, Matsui K (2021). Exacerbation of epilepsy by astrocyte alkalization and gap junction uncoupling. J Neurosci.

[38] Binder DK, Steinhäuser C (2021). Astrocytes and epilepsy. Neurochem Res.

[39] DePew AT, Mosca TJ (2021). Conservation and innovation: versatile roles for LRP4 in nervous system development. J Dev Biol.

[40] Handara G, Hetsch FJA, Jüttner R, Schick A, Haupt C, Rathjen FG, Kröger S (2019). The role of agrin, Lrp4 and MuSK during dendritic arborization and synaptogenesis in cultured embryonic CNS neurons. Dev Biol.

[41] Zhang H, Sathyamurthy A, Liu F, Li L, Zhang L, Dong Z, Cui W, Sun X, Zhao K, Wang H, Ho HH, Xiong WC, Mei L (2019). Agrin-Lrp4-Ror2 signaling regulates adult hippocampal neurogenesis in mice. Elife.

[42] Asadi-Pooya AA, Brigo F, Lattanzi S, Blumcke I (2023). Adult epilepsy. Lancet.

[43] Guerrini R (2006). Epilepsy in children. Lancet.

[44] O'Connor LT, Lauterborn JC, Smith MA, Gall CM (1995). Expression of agrin mRNA is altered following seizures in adult rat brain. Brain Res Mol Brain Res.

[45] Hilgenberg LG, Ho KD, Lee D, O'Dowd DK, Smith MA (2002). Agrin regulates neuronal responses to excitatory neurotransmitters in vitro and in vivo. Mol Cell Neurosci.

[46] Weltha L, Reemmer J, Boison D (2019). The role of adenosine in epilepsy. Brain Res Bull.

[47] Lado FA, Moshé SL (2008). How do seizures stop?. Epilepsia.

[48] Studer FE, Fedele DE, Marowsky A, Schwerdel C, Wernli K, Vogt K, Fritschy JM, Boison D (2006). Shift of adenosine kinase expression from neurons to astrocytes during postnatal development suggests dual functionality of the enzyme. Neuroscience.

[49] Scharbarg E, Daenens M, Lemaître F, Geoffroy H, Guille-Collignon M, Gallopin T, Rancillac A (2016). Astrocyte-derived adenosine is central to the hypnogenic effect of glucose. Sci Rep.

[50] Pagonopoulou O, Efthimiadou A, Asimakopoulos B, Nikolettos NK (2006). Modulatory role of adenosine and its receptors in epilepsy: possible therapeutic approaches. Neurosci Res.

[51] Wang YN, Figueiredo D, Sun XD, Dong ZQ, Chen WB, Cui WP, Liu F, Wang HS, Li HW, Robinson H, Fei EK, Pan BX, Li BM, Xiong WC, Mei L (2018). Controlling of glutamate release by neuregulin3 via inhibiting the assembly of the SNARE complex. Proc Natl Acad Sci USA.

[52] Wang J, Huang J, Li YQ, Yao S, Wu CH, Wang Y, Gao F, Xu MD, Huang GB, Zhao CQ, Wu JH, Zhang YL, Jiao R, Deng ZH, Jie W, Li HB, Xuan A, Sun XD (2021). Neuregulin 1/ErbB4 signaling contributes to the anti-epileptic effects of the ketogenic diet. Cell Biosci.

[53] Wang J, Huang J, Yao S, Wu JH, Li HB, Gao F, Wang Y, Huang GB, You QL, Li J, Chen X, Sun XD (2021). The ketogenic diet increases Neuregulin 1 expression via elevating histone acetylation and its anti-seizure effect requires ErbB4 kinase activity. Cell Biosci.

[54] Racine RJ (1972). Modification of seizure activity by electrical stimulation. II motor seizure. Electroencephalogr Clin Neurophysiol.

[55] Sun XD, Chen WB, Sun D, Huang J, Li YQ, Pan JX, Wang YN, Zhao K, Dong ZQ, Wang HS, Xiong L, Xuan A, Zhao ST, Pillai A, Xiong WC, Mei L (2018). Neogenin in amygdala for neuronal activity and information processing. J Neurosci.

